# Borderline personality disorder and attention deficit/hyperactivity disorder in adolescence: overlap and differences in a clinical setting

**DOI:** 10.1192/j.eurpsy.2023.792

**Published:** 2023-07-19

**Authors:** O. F. Akca, K. Wall, C. Sharp

**Affiliations:** 1 Child and Adolescent Psychiatry, Necmettin Erbakan University Meram School of Medicine, Konya, Türkiye; 2 Psychology, University of Houston, Houston, TX, United States

## Abstract

**Introduction:**

With increased consensus regarding the validity and reliability of diagnosing Borderline Personality

Disorder (BPD) in adolescents, clinicians express concern over the distinction between BPD and Attention-Deficit/Hyperactivity Disorder (ADHD), and its co-morbidity in clinical settings.

**Objectives:**

The goal of this study was to evaluate differences between BPD, ADHD and BPD + ADHD in terms of co-morbid psychiatric disorders and a range of selfreported behavioral problems in adolescents.

**Methods:**

Our sample consisted of N = 550 inpatient adolescents with behavioral and emotional disorders that have not responded to prior intervention. We took a person-centered approach (for increase clinical relevance) and compared adolescents with ADHD, BPD and ADHD+BPD in terms of co-occurring psychiatric disorders and behavioral problems. We performed a regression analysis to test whether BPD symptoms make an incremental contribution to the prediction of psychiatric symptoms over ADHD symptoms.

**Results:**

The severity of almost all co-occurring disorders, aggression, self-harm, suicidal thoughts, and substance use, were higher in the ADHD+BPD group. Borderline symptoms made an incremental contribution to the prediction of psychiatric symptoms beyond the contribution of ADHD.

**Conclusions:**

The findings of this study demonstrated that ADHD and BPD have different psychiatric symptomatology. In addition, subjects who meet criteria for both the BPD and ADHD diagnoses may have more severe psychiatric and behavioral problems compared to individuals with only ADHD or BPD.

**Disclosure of Interest:**

None Declared

